# Twist1 in podocytes ameliorates podocyte injury and proteinuria by limiting CCL2-dependent macrophage infiltration

**DOI:** 10.1172/jci.insight.148109

**Published:** 2021-08-09

**Authors:** Jiafa Ren, Yuemei Xu, Xiaohan Lu, Liming Wang, Shintaro Ide, Gentzon Hall, Tomokazu Souma, Jamie R. Privratsky, Robert F. Spurney, Steven D. Crowley

**Affiliations:** 1 Division of Nephrology, Department of Medicine, Durham VA and Duke University Medical Centers, Durham, North Carolina, USA.; 2 Department of Nephrology, The First Affiliated Hospital of Nanjing Medical University, Nanjing Medical University, Nanjing, China.; 3 Department of Pathology, Nanjing Drum Tower Hospital, The Affiliated Hospital of Nanjing University Medical School, Nanjing, China.; 4 Department of Anesthesiology, Durham VA and Duke University Medical Centers, Durham, North Carolina, USA.

**Keywords:** Nephrology, Chronic kidney disease, Macrophages

## Abstract

The transcription factor Twist1 regulates several processes that could impact kidney disease progression, including epithelial cell differentiation and inflammatory cytokine induction. Podocytes are specialized epithelia that exhibit features of immune cells and could therefore mediate unique effects of Twist1 on glomerular disease. To study Twist1 functions in podocytes during proteinuric kidney disease, we employed a conditional mutant mouse in which Twist1 was selectively ablated in podocytes (Twist1-PKO). Deletion of Twist1 in podocytes augmented proteinuria, podocyte injury, and foot process effacement in glomerular injury models. Twist1 in podocytes constrained renal accumulation of monocytes/macrophages and glomerular expression of CCL2 and the macrophage cytokine TNF-α after injury. Deletion of TNF-α selectively from podocytes had no impact on the progression of proteinuric nephropathy. By contrast, the inhibition of CCL2 abrogated the exaggeration in proteinuria and podocyte injury accruing from podocyte Twist1 deletion. Collectively, Twist1 in podocytes mitigated urine albumin excretion and podocyte injury in proteinuric kidney diseases by limiting CCL2 induction that drove monocyte/macrophage infiltration into injured glomeruli. Myeloid cells, rather than podocytes, further promoted podocyte injury and glomerular disease by secreting TNF-α. These data highlight the capacity of Twist1 in the podocyte to mitigate glomerular injury by curtailing the local myeloid immune response.

## Introduction

Glomerular damage accruing from diverse causes, including diabetes, focal segmental glomerulosclerosis (FSGS), and membranous nephropathy, instigate chronic kidney disease that can progress to end-stage kidney failure (1–4). The pathogenesis of glomerular disease is complex, featuring podocyte injury, a local inflammatory response, and microvascular dysfunction (5, 6). Podocytes are highly specialized, terminally differentiated visceral epithelial cells that maintain the renal filtration barrier. Podocytes are a primary target of injury in a wide range of glomerular diseases marked by consequent albuminuria (7). Renal inflammation is prevalent in proteinuric kidney disease and can trigger or exacerbate podocyte injury (8, 9). Podocytes exhibit characteristic features of immune cells and express components of the innate and adaptive immune cascades that are critical to the inflammatory response in the glomerulus (10–12). For example, pattern recognition receptors including TLRs on podocytes sense pathogen-associated molecular patterns and damage-associated molecular patterns (13), leading to the induction of chemokines and cytokines involved in glomerular damage through the downstream activation of NF-κB signaling (14–16). Elucidating the regulation of immune elements in podocytes should facilitate the development of therapeutic approaches for proteinuric kidney disease.

Transcription of inflammatory response elements such as chemokines and cytokines generally requires transcriptional factor activation or deactivation, making these factors a central regulatory hub of inflammatory gene transcription. Twist1 is a basic helix-loop-helix domain containing transcription factor that governs a wide range of physiologic or pathologic responses that vary based on the cell lineage and the upstream stimulus. For example, Twist1 is a common repressor of cell-mediated and humoral adaptive immunity and limits the immunopathology in inflammatory diseases (17). In T cells, Twist1 negatively regulates proinflammatory gene expression and cytokine production through diminishing NF-κB or Runx3 activation (18–20). In macrophages, Twist1 limits inflammatory responses mainly by suppressing NF-κB activation and cytokine secretion including TNF-α, IL-1β, and IL-6 (21, 22). However, the capacity of Twist1 in podocytes to modulate local immune responses during glomerular damage has not been explored.

In the current study, we examined the role of Twist1 in proteinuric kidney disease. We found that Twist1 expression was induced in glomerular podocytes of renal biopsies from patients with podocytopathies and mice with nephrotoxic serum–induced (NTS-induced) and adriamycin-induced (ADR-induced) glomerular injury. To ascertain the role of Twist1 in podocytes in these diseases, we generated conditional knockout mice in which the *Twist1* gene was selectively disrupted in glomerular podocytes (Twist1-PKO). Deletion of Twist1 in podocytes facilitated glomerular induction of CCL2, which promoted macrophage accumulation and activation, leading to augmented podocyte injury and proteinuria. Accordingly, the inhibition of CCL2 preserved podocyte health and diminished proteinuria, abrogating the differences in these parameters between Twist1-PKO mice and WT controls. Our results suggest that the transcription factor Twist1 protects against podocyte injury by limiting CCL2-dependent macrophage infiltration and activation.

## Results

### Induction of Twist1 expression in podocytes after glomerular injury.

**Figure 1:**
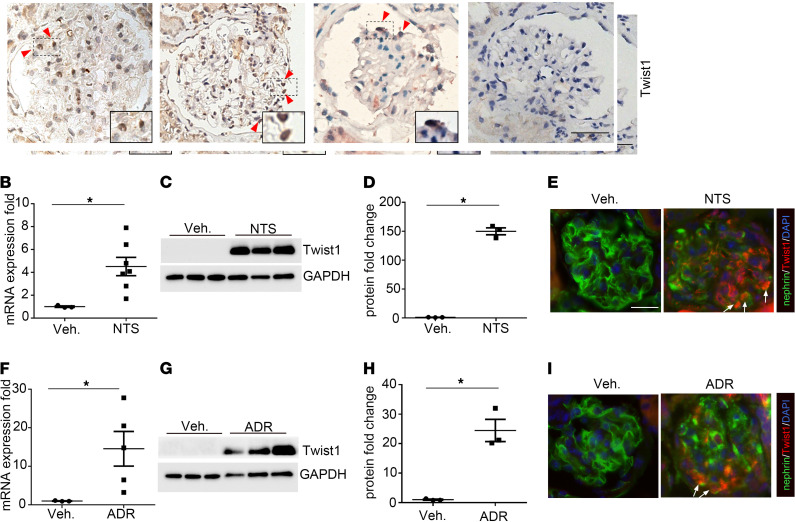
Induction of Twist1 in podocytes from patients with podocytopathies and mice with NTS- or ADR-induced glomerular disease. (**A**) Representative IHC staining of Twist1 in renal biopsies from patients with FSGS, IgA nephropathy, or DN or normal control subjects. Scale bar: 50 μm. (**B** and **C**) Twist1 mRNA (**B**) and protein abundance (**C**) in glomeruli isolated from mice on day 9 after NTS-induced glomerulonephritis (*n* = 3–7, Wilcoxon’s test). (**D**) Semiquantitative determination of Twist1 protein from blots in **C** (*n* = 3, *t* test). (**E**) Representative Twist1- and nephrin-costained kidney sections from vehicle- and NTS-treated animals. (**F** and **G**) Twist1 mRNA (**F**) and protein abundance (**G**) in glomeruli isolated from mice on day 4 after ADR-induced glomerular injury (*n* = 3–7, Wilcoxon’s test). (**H**) Semiquantitative determination of Twist1 protein from blots in **G** (*n* = 3, *t* test). (**I**) Representative Twist1- and nephrin-costained kidney sections from vehicle- and ADR-treated mice. Scale bar: 40 μm. Data represent mean ± SEM. All *t* tests were 2 tailed. *P < 0.05. veh, vehicle; NTS, nephrotoxic serum; ADR, adriamycin; FSGS, focal segmental glomerulosclerosis; DN, diabetic nephropathy.

To investigate the role of Twist1 in proteinuric kidney disease, we first characterized the glomerular expression of Twist1 in renal biopsies from human patients with glomerular damage. IHC staining of these biopsies revealed induction of Twist1 in glomerular podocytes during FSGS, IgA nephropathy, and diabetic nephropathy (DN) compared with controls (Figure 1A). We then confirmed glomerular expression of Twist1 in murine NTS- and ADR-induced nephropathy, 2 widely used models of podocyte injury and proteinuria in mice (23–28). Twist1 mRNA expression was significantly induced in isolated glomeruli after NTS injection (Figure 1B). Consistent with the mRNA induction, Twist1 protein expression in the glomeruli was also significantly upregulated after NTS exposure (Figure 1, C and D). Double-immunofluorescence stains for Twist1 and nephrin suggested that Twist1 expression localized to glomerular podocytes after NTS injection (Figure 1E). The elevation of both Twist1 mRNA and protein expression was similarly detected in the glomerulus during ADR nephropathy (Figure 1, F–I). Thus, Twist1 expression was augmented in the glomerulus during NTS- or ADR-induced podocyte injury.

### Generation of mice with podocyte-specific ablation of Twist1.

**Figure 2:**
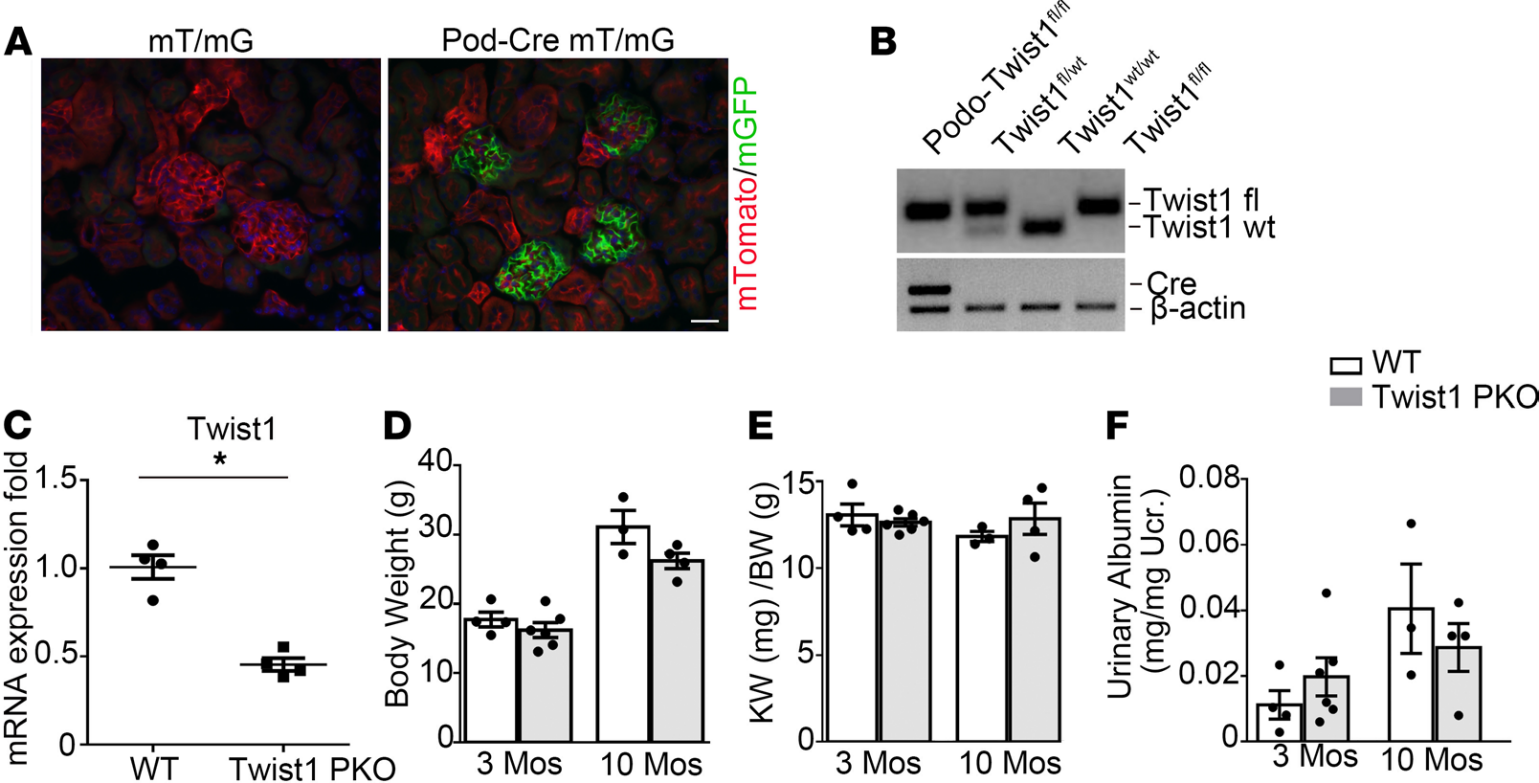
Mice with podocyte-specific ablation of Twist1 are healthy. (**A**) Representative sections of kidneys from Podocin Cre^–^ mT/mG and Podocin Cre^+^ mT/mG reporter mice. Green fluorescence indicates the presence of Podocin Cre expression, whereas red fluorescence indicates the absence of Podocin Cre expression. Blue fluorescence is a nuclear DAPI stain. (**B**) Genotyping of the mice by PCR analysis of genomic DNA. (**C**) Glomerular *Twist1* mRNA expression in Twist1-PKO mice and their control littermates (*n* = 4, *t* test). (**D**–**F**) Body weights (**D**), kidney-to-body weight ratios (**E**), and urinary albumin excretion levels (**F**) of naive Twist1-PKO and WT mice at 3 and 10 months of age (*n* = 3–6, Student-Newman-Keuls test). Data represent mean ± SEM. All *t* tests were 2 tailed. *P < 0.05. Scale bar: 40 μm. KW, kidney weight; BW, body weight; mos, months; Twist1-PKO, Pod-Cre Twist1^fl/fl^.

We generated conditional knockout mice in which the *Twist1* gene was specifically disrupted in glomerular podocytes by using a Cre-Lox gene targeting approach. To confirm the Cre recombinant efficiency in podocytes, we bred an *NPHS2-Cre* mouse line (29) with a double-fluorescence reporter mouse (*mT/mG*). We detected robust Cre expression marked by GFP within the glomeruli of *NPHS2-Cre^+^* (Pod-Cre) *mT/mG* mice but the absence of Cre expression marked by red fluorescent protein in nonglomerular tissues (Figure 2A). We therefore bred the *NPHS2-Cre* mouse line with a *Twist1 flox* line harboring *loxP* sites on either side of the coding region for the *Twist1* gene. For our experiments, we used *NPHS2-Cre*^+^ *Twist1^fl/fl^* mice (Twist1-PKO; Figure 2B, lane 1) and *NPHS2-Cre^–^ Twist1^fl/fl^* (WT; Figure 2B, lane 4) littermates. To confirm podocyte-specific deletion of Twist1 in our Twist1-PKO animals, we isolated glomeruli from Twist1-PKO kidneys. There was a 50% decrease in *Twist1* mRNA expression in PKO-Twist1 glomeruli compared with WT glomeruli (Figure 2C). These results suggest that Twist1 mRNA was also expressed by nonpodocyte glomerular cell lineages, such as mesangial or endothelial cells. There was no difference in body weight, kidney/body weight ratios, and urinary albumin levels between Twist1-PKO and control littermates at 3 and 10 months after birth, respectively (Figure 2, D–F), suggesting that Twist1 was dispensable for maturation and maintenance of a normal glomerular filtration barrier.

### Twist1 limited albuminuria and podocyte marker loss after glomerular injury.

**Figure 3:**
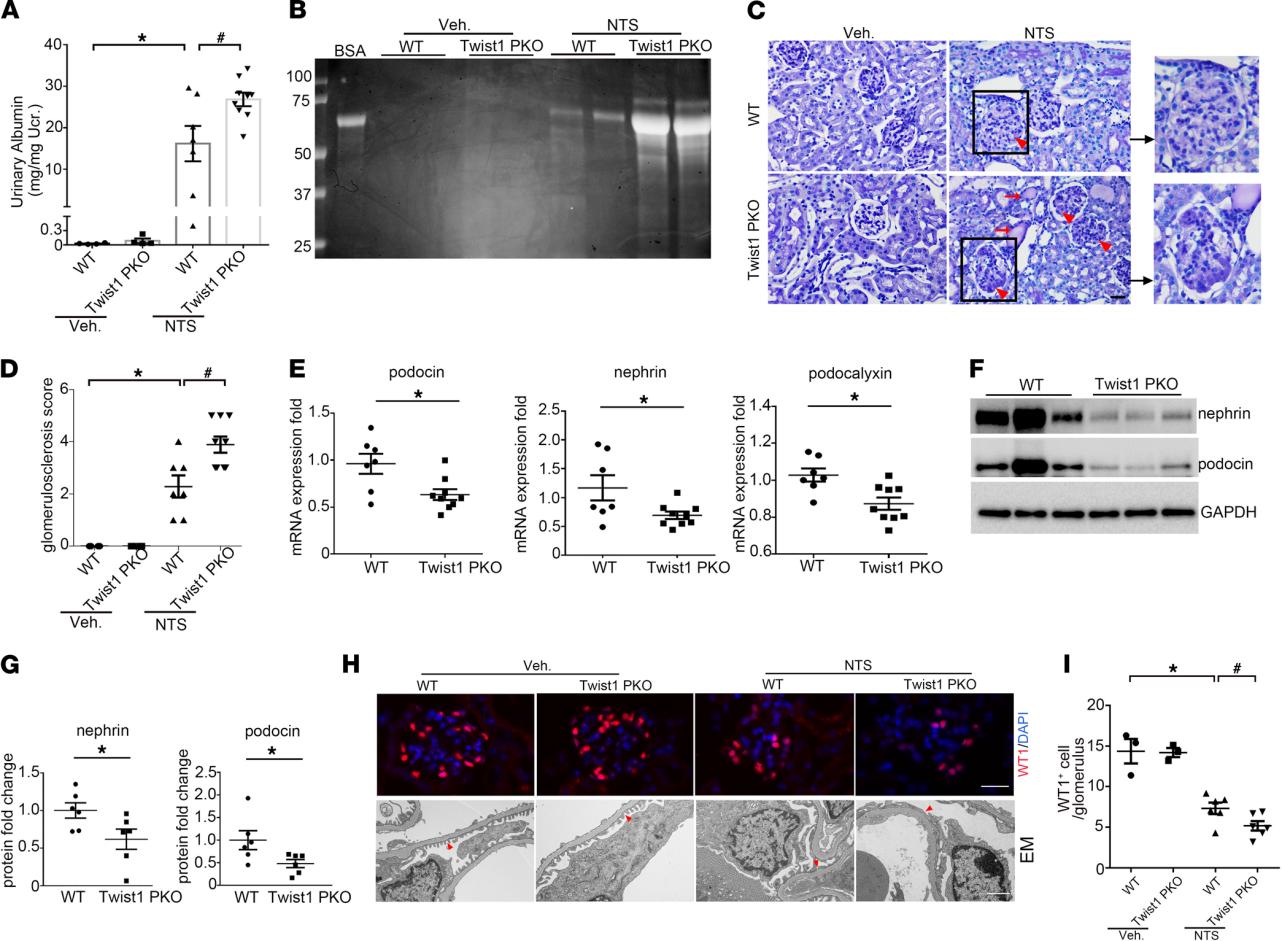
Twist1 in podocytes limits albuminuria and podocyte injury induced by NTS in mice. (**A**) Urinary albumin concentration in WT and Twist1-PKO mice on day 9 vehicle or NTS (*n* = 4–9, Kruskal-Wallis test). (**B**) Representative SDS-PAGE showing the urine proteins in cohorts of mice. (**C**) Representative images of kidney sections from WT and Twist1-PKO mice on day 9 vehicle or NTS. Red arrowheads indicate glomerulosclerosis; red arrows indicate tubular casts. Scale bar: 40 μm. (**D**) Kidney glomerulosclerosis scores in groups (*n* = 4–9, Kruskal-Wallis test). (**E**) Renal mRNA expression of podocin, nephrin, and podocalyxin in WT and Twist1-PKO mice after NTS injection (7–9, *t* test). (**F**) Representative Western blots for nephrin and podocin proteins in glomeruli isolated from WT and Twist1-PKO mice on day 4 NTS. (**G**) Semiquantitative determination of nephrin and podocin protein levels from blots in **F** (*n* = 7–9, *t* test). (**H**) Representative immunostaining for WT1 protein in the groups and electron microscopy showing the glomerular filtration barrier in the groups. (**I**) Quantitative determination of WT1-positive cells in glomeruli in different groups (*n* = 3–6, Student-Newman-Keuls test). Data represent mean ± SEM. All *t* tests were 2 tailed. *P < 0.05, ^#^*P* < 0.05. Red arrowheads indicate foot processes. Scale bar: 40 μm (upper), 1 μm (lower). veh, vehicle; NTS, nephrotoxic serum; EM, electron microscopy; Twist1-PKO, Pod-Cre Twist1^fl/fl^.

The function of podocyte Twist1 was analyzed in the NTS and ADR models of proteinuric kidney disease. On day 9 of NTS, WT mice developed marked albuminuria. Under the same conditions, Twist1-PKO mice developed more severe albuminuria (Figure 3A). Analysis of urine samples by SDS-polyacrylamide gel electrophoresis and Coomasie staining revealed that albumin was the major constituent of urine proteins in mice after NTS injury (Figure 3B). Analysis of renal pathology showed that Twist1-PKO kidneys exhibited more deposition of glomerular matrix compared with WT after NTS (Figure 3, C and D).

We further examined the renal expression of several podocyte markers after NTS. mRNA levels of podocin, nephrin, and podocalyxin were significantly downregulated in NTS kidneys from Twist1-PKO mice compared with WT (Figure 3E). Similarly, at the protein level, nephrin and podocin were expressed at lower levels in Twist1-PKO kidneys (Figure 3, F and G). Accordingly, NTS caused a significant decrease in the number of WT1-positive podocytes in the glomeruli of WT mice, which was exacerbated in the Twist1-PKO mice (Figure 3, H and I). In turn, electron microscopy revealed foot process effacement and loss of the slit diaphragm in some areas of the WT glomeruli after NTS, with more severe disruptions in podocyte architecture in the Twist1-PKO kidneys (Figure 3H).

**Figure 4:**
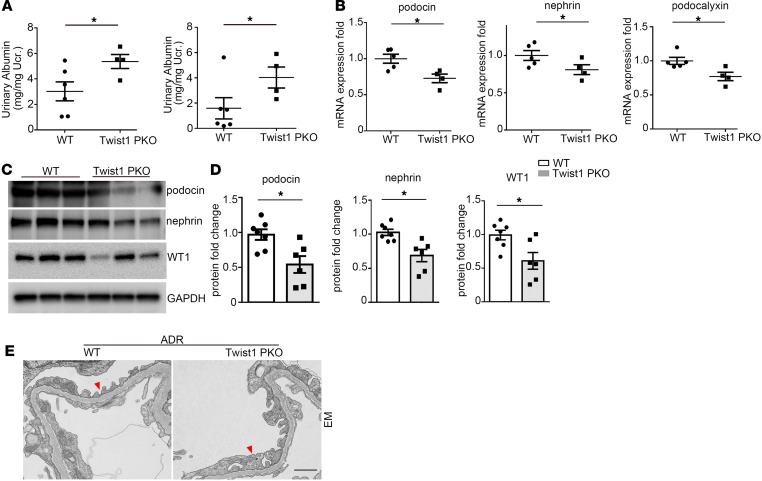
Twist1 in podocytes mitigates albuminuria and podocyte injury induced by ADR in mice. (**A**) Urinary albumin concentration in WT and Twist1-PKO mice on day 1 (left) and day 4 (right) ADR (*n* = 4–6, *t* test). (**B**) Renal mRNA expression of podocin, nephrin, and podocalyxin in WT and Twist1-PKO mice after ADR injection (*n* = 4–5, *t* test). (**C**) Western blot for podocin, nephrin, and WT1 protein in glomeruli isolated from WT and Twist1-PKO mice after ADR injection. (**D**) Semiquantitative determination of podocin, nephrin, and WT1 protein from blots in **C** (6–7, *t* test). (**E**) EM shows glomerular filtration barrier in different groups after ADR exposure. Red arrowheads indicate foot processes. Scale bar: 1 μm. Data represent mean ± SEM. All *t* tests were 2 tailed. *P < 0.05. ADR, adriamycin; EM, electron microscopy; Twist1-PKO, Pod-Cre Twist1^fl/fl^.

To confirm these findings in a separate model of glomerular injury, WT and Twist1-PKO animals were subjected to ADR nephropathy. ADR-treated Twist1-PKO mice exhibited significant increases in urinary albumin excretion (Figure 4A; see Figure 3A for baseline levels). Kidneys from these animals showed significant decreases in podocin, nephrin, and podocalyxin at the mRNA level (Figure 4B) and podocin, nephrin, and WT1 at the protein level (Figure 4, C and D) compared with ADR-treated WT controls. Here again, electron microscopy analysis revealed more severe lesions in the foot processes and slit diaphragms of the Twist1-PKO glomeruli compared with WT after ADR (Figure 4E). These data suggest that Twist1 in podocytes ameliorated proteinuria and podocyte injury in both the NTS and the ADR glomerular disease models.

Because urinary albumin can be reabsorbed in the nephron, leading to tubulointerstitial damage, we examined whether deletion of Twist1 in podocytes influenced injury in the tubular compartment. To this end, we blindly scored levels of tubular damage in WT and Twist1-PKO mice subjected to NTS. As in the glomerulus, Twist1-PKO animals compared with WT showed more severe tubular injury (Supplemental Figure 1A; supplemental material available online with this article; https://doi.org/10.1172/jci.insight.148109DS1), corroborated by higher renal mRNA levels of the *Havcr1* gene encoding the tubular injury marker Kim-1 (Supplemental Figure 1B). We also noted exaggerated renal mRNA expression for mediators of interstitial fibrosis, including TGF-β, α–smooth muscle actin, and plasminogen activator inhibitor 1 (Supplemental Figure 1C). However, in these acute glomerular injury models, we did not detect differences in interstitial fibrosis (data not shown). Although Twist1 is known to regulate gene expression programs for epithelial-mesenchymal transition (EMT), mRNA expression levels for the EMT markers Snail and Slug were similar in the 2 groups. By contrast, mRNA for E-cadherin was downregulated to a greater degree in the Twist1-PKO cohort (Supplemental Figure 1D). All of these findings from the tubular compartment were largely recapitulated in the ADR model (Supplemental Figure 1, E–G). Lastly, we did not detect significant differences between the groups in markers of kidney function, serum blood urea nitrogen and creatinine, in either model (Supplemental Figure 2, A–D).

### Twist1 in podocytes limited CCL2 and TNF-α induction in injured glomeruli.

**Figure 5:**
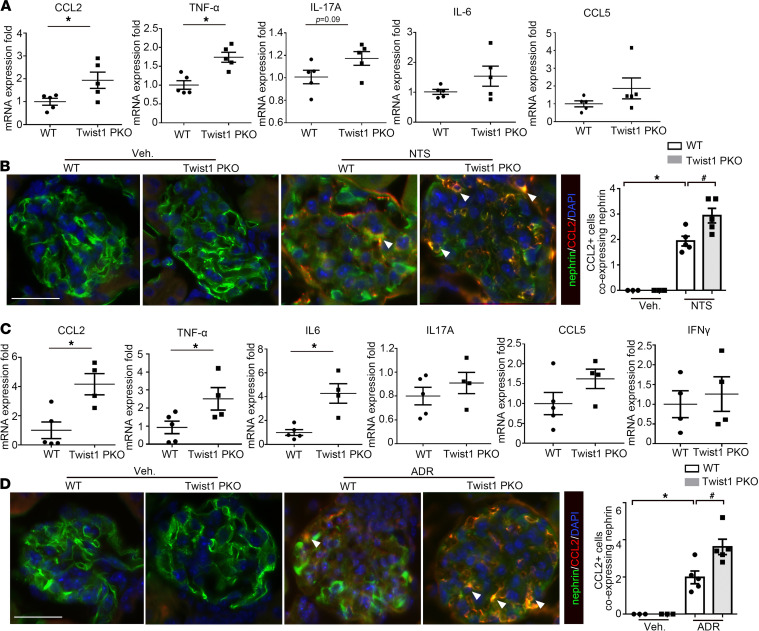
Twist1 in podocytes suppresses CCL2 and TNF-α mRNA expression in isolated glomeruli after NTS or ADR injury. (**A**) mRNA expression for CCL2, TNF-α, IL-17A, IL-6, and CCL5 in isolated Twist1-PKO and WT glomeruli after in vivo NTS exposure (*n* = 4–5, *t* test). (**B**) Representative costaining for nephrin and CCL2 protein in the glomeruli after in vivo NTS exposure and quantitative determination of CCL2-positive cells expressing nephrin in glomeruli (*n* = 3–5, Student-Newman-Keuls test). (**C**) mRNA expression for CCL2, TNF-α, IL-6, IL-17A, CCL5, and IFN-γ in isolated Twist1-PKO and WT glomeruli after in vivo ADR injection (*n* = 4–5, *t* test). (**D**) Representative costaining for nephrin and CCL2 protein in the groups after in vivo ADR exposure and quantitative determination of CCL2-positive cells expressing nephrin in glomeruli (*n* = 3–5, Student-Newman-Keuls test). All *t* tests were 2 tailed. *P < 0.05, ^#^*P* < 0.05. Data represent mean ± SEM. White arrowheads indicate double-positive cells. Scale bar: 40 μm. NTS, nephrotoxic serum; ADR, adriamycin; Twist1-PKO, Pod-Cre Twist1^fl/fl^.

Twist1 plays a key role in modulating chemokine and proinflammatory cytokine production. To analyze the downstream effects of podocyte Twist1 on inflammatory mediators in the glomerulus, we isolated glomeruli from Twist1-PKO and WT kidneys after injury and measured mRNA expression for a panel of cytokines and chemokines relevant to glomerular damage by quantitative PCR (30, 31). After NTS, glomeruli from mice with Twist1 deficiency in podocytes had increased levels of CCL2 and TNF-α compared with WT (Figure 5A). We therefore posited that Twist1 in podocytes may protect the glomerulus by suppressing local generation of CCL2 and/or TNF-α. To delineate which cells express CCL2 after NTS exposure, we carried out double immunostaining for CCL2 and the podocyte marker nephrin. As shown in Figure 5B, most CCL2 protein colocalized with nephrin protein, suggesting podocytes may have been a key source of CCL2 after NTS exposure. These findings were recapitulated after ADR-induced injury (Figure 5, C and D). To determine if TNF-α from podocytes impacts glomerular injury, we generated conditional knockout mice in which TNF-α was specifically deleted in podocytes (TNF-PKO) using the Cre-Lox system. As shown in Supplemental Figure 3, A–D, we found no differences in either albuminuria or glomerular structure between the WT and TNF-PKO groups after NTS- or ADR-induced injury. Because our previous studies have supported a role for TNF-α from macrophages in proteinuria and glomerulosclerosis (32), the current experiments suggest that TNF-α derived from recruited macrophages, rather than podocytes, contributed to podocyte injury.

### Twist1 constrained CCL2 generation in cultured podocytes in vitro.

**Figure 6:**
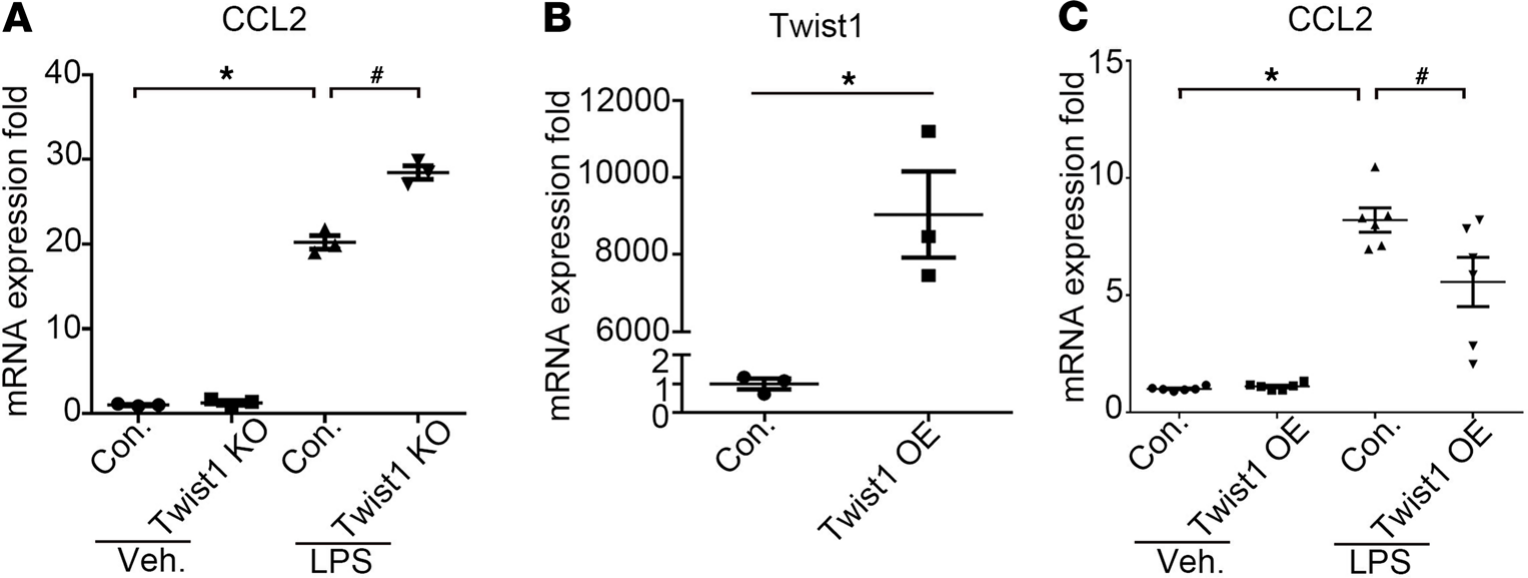
Twist1 deletion in podocytes enhances LPS-induced CCL2 expression. (**A**) mRNA expression for CCL2 in primary WT and Twist1-KO podocytes stimulated with LPS (*n* = 3, Student-Newman-Keuls test). (**B**) mRNA expression for Twist1 in primary podocytes transfected with Twist1-OE or control plasmid (*n* = 3, *t* test). (**C**) mRNA expression for CCL2 in primary WT and Twist1-OE podocytes stimulated with LPS (*n* = 6, Student-Newman-Keuls test). All *t* tests were 2 tailed. *P < 0.05, ^#^*P* < 0.05. con, control plasmid; OE, overexpressing plasmid; veh, vehicle.

As a complementary approach, we next examined whether Twist1 could regulate CCL2 generation in podocytes in vitro. As TLR4 is expressed on podocytes and is upregulated in glomerular disease (13, 15), we isolated and cultured primary podocytes from WT and Twist1-PKO animals and then stimulated TLR4 on the podocytes with LPS for 6 hours to mimic the podocyte activation that occurs during inflammatory injury (15). In WT podocytes, LPS caused dramatic induction of CCL2 mRNA, an effect that was enhanced in Twist1-KO podocytes (Figure 6A). Inversely, we explored whether the addition of exogenous Twist1 could limit the expression of CCL2 in cultured WT podocytes. To test this possibility, we transfected primary mouse podocytes with Twist1-overexpressing plasmid or control. In podocytes transfected with Twist1, *Twist1* mRNA was markedly elevated compared with levels in podocytes transfected with control plasmid (Figure 6B). In turn, the overexpression of Twist1 reduced levels of CCL2 in LPS-treated podocytes (Figure 6C). By contrast, we could not detect an effect of Twist1 deletion or overexpression in LPS-treated podocytes on mRNA expression for the EMT markers Snail or Slug (Supplemental Figure 4, A and B), the podocyte markers nephrin or podocin, (Supplemental Figure 4C), or the inflammatory cytokines IL-6, TNF-α, or IL-17A (Supplemental Figure 4, D–F). Twist1-dependent expression changes for CCL5 in the podocytes mimicked those of CCL2 (Supplemental Figure 4G). To determine if Twist1 suppresses CCL2 or CCL5 expression in podocytes via direct interaction with their promoters, we performed ChIP analysis with vehicle- or LPS-treated WT podocytes. We could not find evidence for direct interactions of Twist1 with the promoters for the genes encoding CCL2 or CCL5 (Supplemental Figure 4H). These in vitro experiments corroborated our in vivo findings that Twist1 in podocytes limited CCL2 generation after glomerular injury.

### Twist1 in podocytes suppressed the accumulation of proinflammatory monocytes/macrophages in the injured glomerulus.

**Figure 7:**
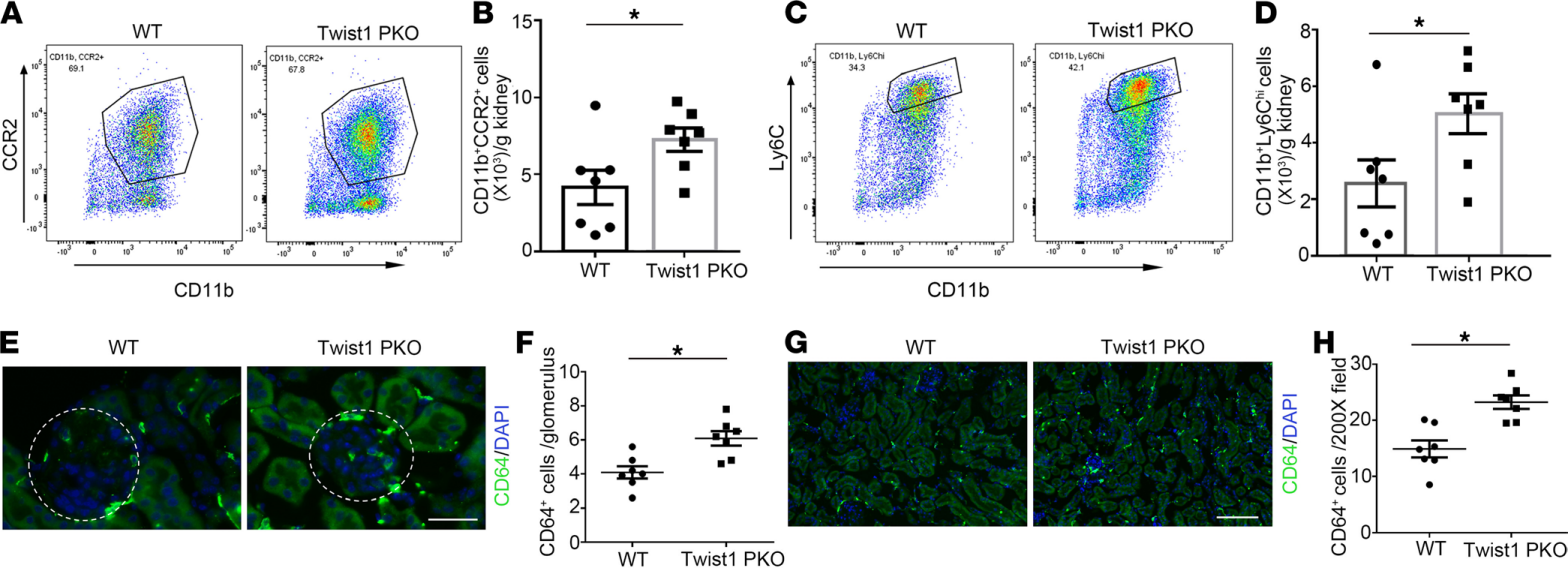
Twist1 in podocytes suppresses macrophage accumulation in glomeruli and renal interstitium after NTS injury. (**A**) Representative flow plots of CD11b^+^CCR2^+^ macrophages from kidneys of NTS-treated WT and Twist1-PKO animals. (**B**) Absolute numbers of CD11b^+^CCR2^+^ macrophages per gram of WT or Twist1-PKO kidney (*n* = 7, *t* test). (**C**) Representative flow plots of CD11b^+^Ly6C^hi^-infiltrating monocytes from kidneys of NTS-treated WT and Twist1-PKO animals. (**D**) Absolute numbers of CD11b^+^Ly6C^hi^-infiltrating monocytes per gram of WT and Twist1-PKO kidney (*n* = 7, *t* test). (**E**) Representative sections of CD64-stained glomeruli from NTS-treated WT and Twist1-PKO animals. Scale bar: 40 μm. (**F**) Quantitative determination of glomerular CD64-positive cells in NTS-treated WT and Twist1-PKO cohorts (*n* = 7, *t* test). (**G**) Representative CD64-stained kidney sections from NTS-treated WT and Twist1-PKO mice. Scale bar: 100 μm. (**H**) Quantitative determination of kidney interstitial CD64-positive cells in NTS-treated WT and Twist1-PKO cohorts (*n* = 7, *t* test). All *t* tests were 2 tailed. *P < 0.05. NTS, nephrotoxic serum; Twist1-PKO, Pod-Cre Twist1^fl/fl^.

CCL2 activates CCR2 on proinflammatory monocytes/macrophages and draws them into inflamed glomeruli, where myeloid cells play a vital role in podocyte injury and glomerulosclerosis. To explore if the regulation of CCL2 by Twist1 in podocytes could be important in modulating glomerular damage, we examined renal macrophage accumulation in our WT and Twist1-PKO cohorts after NTS-induced injury. We first isolated infiltrating CD45^+^ leukocytes from the injured kidney via flow cytometry and quantified the number of CD11b^+^CCR2^+^Ly6G^–^ monocytes/macrophages (Supplemental Figure 5). The number of CCR2^+^ monocytes/macrophages from Twist1-PKO kidneys was higher than from WT (Figure 7, A and B). Previous studies have revealed that circulating proinflammatory monocytes expressing Ly6C infiltrate injured organs via a CCR2-dependent mechanism and then differentiate into macrophages. Accordingly, Twist1-PKO kidneys contained greater numbers of Ly6C^hi^ myeloid cells than WT controls (Figure 7, C and D).

To analyze whether podocyte Twist1 influenced the accumulation of macrophages in the glomerulus or renal interstitium, we quantified macrophage accumulation in these compartments by immunohistology. Immunofluorescence staining for the macrophage marker CD64^+^ in the glomeruli showed augmented numbers of glomerular macrophages in the Twist1-PKO mice compared with WT (Figure 7, E and F), which could have contributed to the exaggerated podocyte injury and glomerulosclerosis in the Twist1-PKO animals. Similarly, the accumulation of interstitial macrophages in Twist1-PKO kidneys was enhanced compared with that in WT controls (Figure 7, G and H). Thus, Twist1 in podocytes limited the recruitment of proinflammatory monocytes into the glomerulus and interstitium during NTS injury, possibly through a CCL2-dependent mechanism.

### CCR2 inhibition ameliorated albuminuria and rescued the expression of podocyte markers in Twist1-PKO mice after NTS injury.

**Figure 8:**
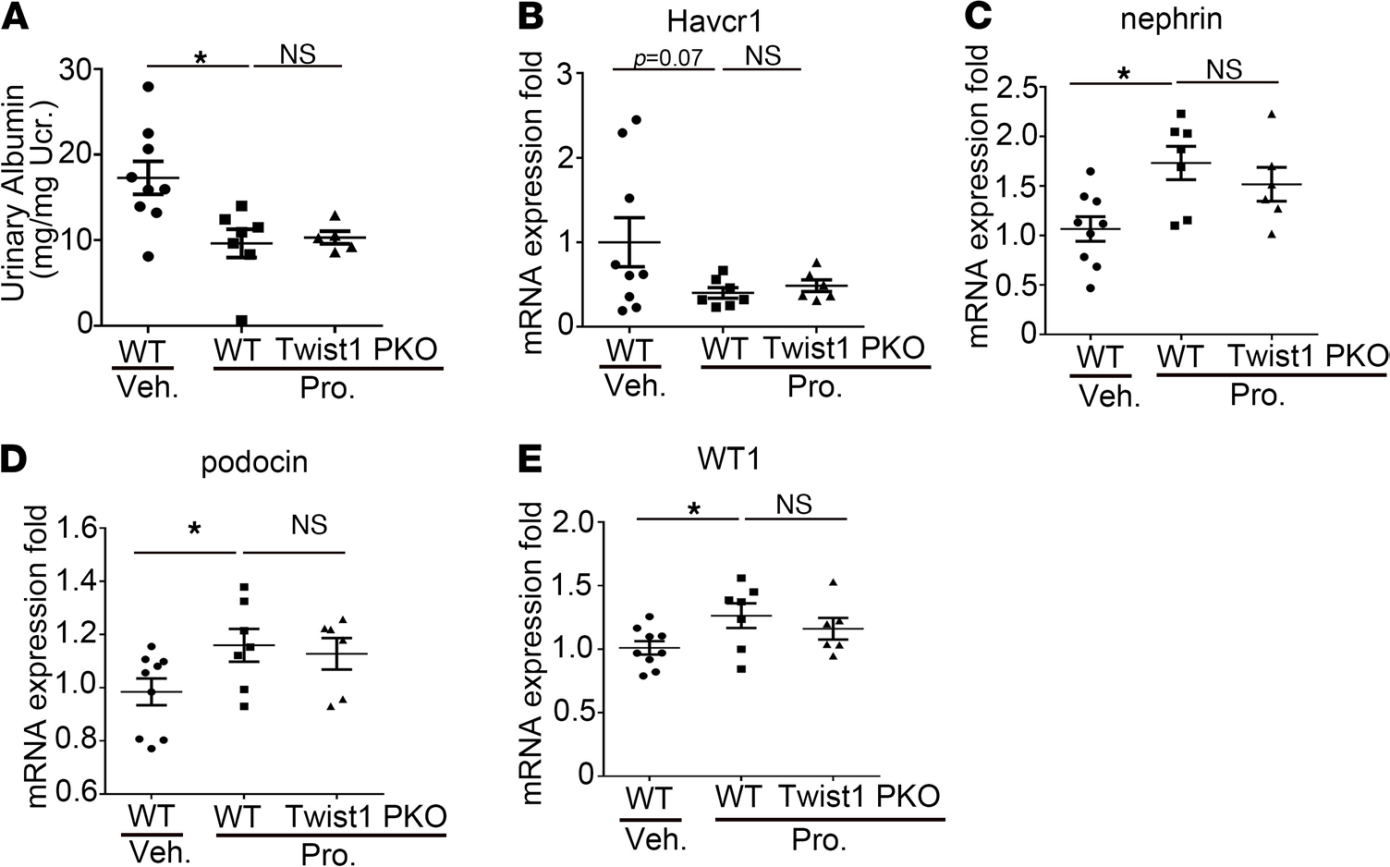
CCL2 inhibition abrogates exaggerated albuminuria and restores podocyte marker expression in Twist1-PKO animals after NTS injury. (**A**) Urinary albumin concentration in groups after NTS injury (*n* = 6–9, Student-Newman-Keuls test). (**B**–**E**) mRNA expression for Kim-1 (**B**), nephrin (**C**), podocin (**D**), and WT1 (**E**) in kidney tissues from groups as indicated (*n* = 6–9, Student-Newman-Keuls test). *P < 0.05. NTS, nephrotoxic serum; pro, propagermanium; Twist1-PKO, Pod-Cre; Twist1^fl/fl^.

Finally, to directly test whether the augmented glomerular induction of CCL2 permitted by Twist1 deficiency in podocytes exacerbates albuminuria and podocyte injury, we treated WT and Twist1-PKO animals with the CCL2 inhibitor propagermanium during NTS. CCL2 inhibition reduced albuminuria and mRNA levels for *Havcr1* gene encoding the kidney injury marker Kim-1 in the WT mice and equalized levels of these parameters in the WT and Twist1-PKO cohorts (Figure 8, A and B). Inversely, the CCL2 antagonist preserved mRNA levels for podocyte differentiation markers nephrin, podocin, and WT1 in the WT animals and abrogated differences in expression for these genes between the WT and Twist1-PKO cohorts (Figure 8, C–E). Similarly, the CC2 antagonist preserved podocyte numbers in the WT glomeruli and equalized podocyte numbers between the WT and Twist1-PKO cohorts (Supplemental Figure 6). Thus, CCL2 inhibition ameliorated albuminuria and podocyte loss and injury permitted by Twist1 deficiency in podocytes after an NTS insult. These data suggest that Twist1 protected the podocyte by limiting CCL2-dependent macrophage accumulation in the injured glomerulus.

## Discussion

Twist1 is a widely expressed transcription factor that broadly impacts both tubular epithelial cell dedifferentiation after renal injury and cellular immune responses during chronic disease (17, 20, 21, 33). Accordingly, targeting of the Twist1 signaling pathway may be challenged by the divergent effects of Twist1 in renal tubular cells and leukocytes on the progression of kidney damage (33–35). Because the podocyte is a primary target of injury during glomerular disease and exhibits features of both epithelial and immune cell lineages, we explored the impact of Twist1 in podocytes on glomerular damage after immune-mediated or toxic insult. We find that Twist1 was upregulated in the damaged glomerulus in human kidney disease and murine models and that Twist1 in the murine podocyte limited CCL2-dependent macrophage accumulation, podocyte loss, and albuminuria. Thus, Twist1 protected the podocyte through an immune-mediated mechanism.

Podocytes produce a variety of factors to mediate cellular communication with other cell lineages present in the healthy or injured glomerulus. For example, in normal conditions, podocytes secrete VEGF (36, 37), angiopoietin-1 (38), and CXCL12 (39) to regulate the vascular development or maintain endothelial homeostasis. In pathologic conditions, the podocyte-specific overexpression of VEGF drives collapsing glomerulopathy (37). The chemokine CCL21 secreted by podocytes interacts with CCR7 on mesangial cells to regulate mesangial cell proliferation and migration (40), and podocyte-derived PDGF similarly mediates mesangial cell proliferation and glomerulosclerosis (41). Our current studies demonstrate that Twist1-regulated CCL2 generation played a pivotal role in crosstalk between podocytes and macrophages during the development of podocyte injury and glomerulopathy.

Our experiments indicate that Twist1 in podocytes suppressed their generation of the macrophage chemokine CCL2 and thereby constrained the accumulation of macrophages in injured glomeruli. These infiltrating macrophages then propagated podocyte dysfunction and injury by secreting TNF-α (31, 32). Twist1 could have regulated CCL2 production through multiple mechanisms. Twist1 modulates activity of NF-κB, a master regulator of inflammatory signaling pathways, and blunts the cellular response to cytokine-induced NF-κB activation through a negative feedback loop (14, 17, 21). For example, in white adipose tissues, low Twist1 expression associates with higher levels of proinflammatory cytokines. Twist1 silencing enhances TNF-α–induced CCL2 expression and secretion, possibly by repressing RelA/p65-mediated transcriptional activation of several cytokine promoters (42). We could not detect evidence of direct interactions between Twist1 and CCL2’s promoter, so the effects of Twist1 on CCL2 expression in podocytes may be indirect as reported in adipocytes (43). Additionally, Twist1 is an upstream regulator of GPS2 and SMRT, both of which are associated with repression of CCL2 expression (43).

CCL2 expression localizes prominently to glomerular podocytes in several human and experimental proteinuric kidney diseases, including diabetic, hypertensive, membranous, lupus, and crescentic nephropathy (16, 44–48). Urinary CCL2 can be used as a biomarker of kidney inflammation (49). In a group of patients with chronic kidney disease, the levels of albuminuria correlated closely with urinary CCL2 excretion (50). CCL2 can injure podocytes indirectly by attracting macrophages and promoting inflammation (30) but can also have direct effects on podocytes. The ligation of CCR2 on podocytes stimulates their motility (51), with possible effects on the progression of crescentic glomerulonephritis (52). CCL2 can modulate the structure of the podocyte actin cytoskeleton with consequent effects on the slit diaphragm’s permeability to albumin (51).

Through the ligation of CCR2, CCL2 attracts circulating monocytes/macrophages into sites of injury and promotes differentiation of these myeloid cells toward a proinflammatory “M1” phenotype (53). Persistent M1 macrophage infiltration and associated inflammation can play critical pathogenic roles in driving podocyte dysfunction and glomerular disease. Accordingly, macrophage deletion or disruption of macrophage infiltration ameliorates podocyte injury, albuminuria, and glomerular pathology (24, 54). Specifically, blocking CCR2’s functions limits the progression of CKD in several models (53, 55). We therefore distinguished monocyte/macrophage populations in the NTS kidneys by detecting levels of CCR2 expression on these myeloid cells. In line with the more severe glomerular injury in the Twist1-PKO kidneys, we found markedly higher numbers of CCR2^+^ proinflammatory myeloid cells in the Twist1-PKO cohort. We also measured renal myeloid cell expression of Ly6C, another key proinflammatory, bone marrow–derived monocyte surface marker (56, 57). Here again, more CD11b^+^Ly6C^hi^ myeloid cells infiltrated the kidneys of Twist1-PKO animals than in the WT cohort after NTS. Because deletion of TNF-α in podocytes did not impact podocyte injury in the current studies, we conclude that TNF-α produced by proinflammatory macrophages, rather than podocytes, plays a fundamental role in driving glomerular disease (31, 32). Collectively, our data suggest that Twist1 in podocytes mitigates glomerular injury by restricting CCL2-dependent accumulation of TNF-α–expressing macrophages in the glomerular compartment (58–60). We acknowledge that the data in our models reflect acute damage to the glomerulus and do not capture more chronic changes in glomerular architecture.

In summary, Twist1 is a transcription factor that modulates expression of CCL2 in the kidney glomerulus. During glomerular disease, Twist1 in podocytes limited CCL2-induced recruitment of monocytes/macrophages into the glomerulus and thereby mitigated damage driven by TNF-α–elaborating proinflammatory macrophages. Thus, Twist1 was a key regulator of crosstalk between podocytes and myeloid cells within the glomerulus. Through this crosstalk, Twist1 in podocytes ameliorated podocytopathy and restrained glomerular disease progression. Our findings underscore the potential for Twist1 as a promising target for cell-specific therapeutic modalities in the treatment of proteinuric kidney diseases.

## Methods

### Animals.

Homozygous *Twist1-*floxed mice on a 129/SvEv background (a gift from Richard Behringer, Department of Genetics, Division of Basic Sciences, MD Anderson Cancer Center, Houston, Texas, USA) (61) were crossed with 129/SvEv *Nphs2-Cre* mice (29) to generate *Nphs2-Cre^+^ Twist1^fl/fl^* (Twist1-PKO) and WT littermates. Similarly, 129/SvEv *TNF^fl/fl^* mice (62) were bred with 129/SvEv *Nphs2-Cre* mice to yield *Nphs2-Cre^+^ TNF^fl/fl^* mice and WT control subjects. To map the distribution pattern of *Cre* recombinase expression in kidney, *mT/mG* mice from The Jackson Laboratory were crossed with the *Nphs2-Cre* recombinase transgenic lines. mT/mG mice normally express red fluorescent protein in all tissues. When *Cre* is present, the mT cassette is deleted, triggering expression of membrane-targeted *eGFP*. Female and male mice aged 8–12 weeks were used for NTS and ADR models, respectively.

### Human kidney biopsy specimens.

Human kidney specimens were obtained from diagnostic renal biopsies performed in the Affiliated Hospital of Nanjing University Medical School. Nontumor kidney tissues from the patients who had renal carcinoma and underwent nephrectomy were used as the normal controls. Paraffin-embedded human kidney biopsy sections were prepared using a routine procedure. Kidney biopsy specimens were then stained with Twist1 (Abcam, catalog 50887) antibody.

### Histological analysis and immunofluorescence staining.

Mouse kidney samples were fixed in 10% formalin (MilliporeSigma) overnight and embedded in paraffin. Paraffin sections (5 μm thick) were stained by the periodic acid–Schiff reagent, and the histologic changes were assessed by light microscopy. An investigator who was blinded to experimental conditions scored at least 20 random glomeruli under the microscope. A score of 0 represents the percentage of sclerotic glomeruli less than 5%, whereas 1, 2, 3, 4, and 5 connote 6%–20%, 21%–40%, 41%–60%, 61%–80, and greater than 81% of the selected glomeruli, respectively. A score was assigned to each mouse. The score for tubular injury including dead tubules, loss of brush borders, tubule dilatation, cast formation, tubular epithelial swelling, and vacuolar degeneration was semiquantitative. A score of 0 represents injury area less than 5%, whereas 1, 2, 3, and 4 exhibit damage involving 5%–25%, 26%–50%, 51%–75%, and greater than 75% of the whole kidney area, respectively. At least 10 random fields were assessed under the microscope (original magnification, ×200). A tubular injury score was assigned to each mouse.

The frozen kidney sections were blocked with 1% BSA containing 0.3% Triton X-100 for 30 min and then incubated with Twist1 (Abcam, catalog 50887),WT1 (Abcam, catalog 89901), nephrin (Fitzgerald, catalog 20R-NP002), CD64 (Thermo Fisher Scientific, catalog MA5-29706), or CCL2 (Abcam, catalog ab8101) primary antibody at 4°C overnight. After washing off the primary antibody, sections were incubated for 1 hour at room temperature with respective secondary antibodies (Thermo Fisher Scientific, catalog A-21203, PA1-28593, A-21207, A-21209, or A-21206). After washing 3 times with PBS, the slides were mounted and visualized by fluorescence microscopy.

### Animal models of podocyte injury and proteinuria.

The mouse models for podocyte injury and proteinuria were established by i.v. injection of NTS or ADR. For NTS, mice received an i.p. injection of sheep IgG (250 μg/mouse) on day 0. Tail injection of sheep NTS (catalog PTX-001S, Probetex) was performed on day 5. On day 9, mice were euthanized, and urine and kidney tissues were harvested for further analysis. Mice were administered ADR (18 mg/kg) by i.v. tail vein injection. Urine was collected on day 1 and day 4, and all mice were euthanized and kidneys were collected on day 4 after ADR exposure.

### Glomerulus isolation.

Mouse glomeruli were isolated according to a method described elsewhere (63). Briefly, mice were anesthetized with isoflurane and perfused through the heart, with HBSS containing 2.5 mg/mL iron oxide (MilliporeSigma) and 0.1% BSA. After perfusion, the kidneys were removed, cut into 1 mm^3^ pieces, and digested in HBSS containing 1 mg/mL collagenase A and 100 U/mL deoxyribonuclease I. Digested tissue was then passed through a 100 μm cell strainer and collected by centrifugation at 350*g* for 5 minutes at 4°C. The pellet was resuspended in 2 mL of HBSS, and the glomeruli were collected using a magnet. The purity of glomeruli was verified under microscopy and attained more than 95% by this method (Supplemental Figure 3E). The isolated glomeruli were used for subsequent RNA or protein analysis.

### Mouse primary podocyte culture and treatment.

Primary podocyte culture was performed according to a method described elsewhere with minor modifications (64). The glomerular isolation procedure was according to the method described above. The isolated glomeruli were placed in collagen I–coated (MilliporeSigma) culture dishes and cultured in RPMI 1640 (Gibco, Thermo Fisher Scientific) plus 10% FBS at 37°C. Subculture of primary cultured podocytes was performed after 4–5 days of culture of isolated glomeruli. Cellular outgrowths were detached with trypsin (MilliporeSigma) and passed through a 25 μm sieve to remove the remaining glomerular cores. The filtered cells were cultured on collagen I–coated dishes for one more day and then processed for subsequent studies. Twist1 plasmid (pBABE-puro-mTwist, catalog 1783, Addgene) or control plasmid (pBABE-puro-EGFP, catalog 128041, Addgene) was transfected into primary podocytes using Lipofectamine 3000 reagent (catalog L3000015, Thermo Fisher Scientific) according to the manufacturer’s instructions. Cells were then incubated with LPS (1 mg/mL) for 6 hours to induce an inflammatory response.

### Urinary albumin measurement.

The levels of urinary albumin and creatinine were measured with kits from Exocell (catalog 1011 and catalog 1012, respectively, Ethos Biosciences) according to instructions. Urinary albumin excretion was expressed as the albumin/creatinine ratio. Urinary proteins were also analyzed by SDS-polyacrylamide gel electrophoresis after normalization to urinary creatinine. After separation by SDS-polyacrylamide gel electrophoresis, urine proteins were stained with Coomassie blue R-250.

### Western blot analysis.

Protein expression was detected by Western blot analysis as described previously (35). Briefly, the isolated glomeruli were homogenized in RIPA buffer (MilliporeSigma). The supernatants were collated after centrifugation at 13,000*g* at 4°C for 30 minutes. Concentrations of protein were quantitated using the DC Protein Assay Kit (Bio-Rad). Equal amounts of sample were subjected to electrophoresis through 4%–12% Bis-Tris gels and transferred to polyvinylidene difluoride membranes. After blocking with 5% milk in Tris-buffered saline/Tween 20, the blots were incubated with anti-Twist1 (Abcam, catalog 50887), anti-nephrin (Thermo Fisher Scientific, catalog PA5-91907), anti-podocin (MilliporeSigma, catalog P0372), or anti-GAPDH (Cell Signaling Technology [CST], catalog 2118) overnight at 4°C. The blots were then washed and incubated for 1 hour at room temperature with respective secondary antibodies accordingly (CST, catalog 7074 and 7076, or Abcam, catalog ab6741). Bands were detected using an enhanced chemiluminescence detection system (BiO-RAD ChemiDoc MP Imaging System). The detected bands were quantified by densitometry through ImageJ 1.38 for Windows (NIH).

### Kidney flow cytometry.

Kidney single-cell suspensions were prepared by mechanical and enzymatic digestion as described previously (34, 35). The kidney single-cell suspensions were incubated with Fc Block (BioLegend) for 30 minutes and treated with anti-CD45 (BioLegend, 103139), anti-Ly6G (BD Biosciences, 560603), anti-CD11b (BD Biosciences, 552850), anti-CCR2 (R&D Systems, Bio-Techne, FAB8368P), anti-Ly6C (BD Biosciences, 560596), and near-infrared dead cell indicator (Life Technologies, L34976) for 30 minutes at 4°C. Cells were washed and fixed with Fix/Perm buffer (catalog 554655, BD Biosciences). Then 20 μL of CountBright absolute counting beads (Invitrogen, Thermo Fisher Scientific, catalog C36950) were added to cells, and samples were analyzed on a BD LSR II flow cytometer. Data were analyzed using FlowJo software version 10.2 (Tree Star, Inc.). Absolute CD11b^+^CCR2^+^Ly6G^–^ and CD11b^+^Ly6C^+^Ly6G^–^ cell numbers from each sample were obtained according to CountBright’s manufacturer’s instructions. The detailed gating strategy is shown in Supplemental Figure 5.

### ChIP.

Primary podocytes were cultured in 15 cm plates to 90% confluence and treated with vehicle or LPS (1 mg/mL). ChIP assays were performed according to the manufacturer’s instructions (CST, catalog 9003). Anti-Twist1 antibody (Abcam, catalog 50887) and anti–histone H3 (CST, catalog 4620) as a positive control antibody and IgG as a negative control were used in the ChIP assay. The primers were designed at multiple locations within 2000 bp upstream relative to the transcription start site of the genes encoding CCL2 or CCL5.

### CCR2 inhibitor treatment experiments.

Twist1-PKO and WT mice received the CCR2 inhibitor, propagermanium (8 mg/kg/d), or vehicle by oral gavage beginning on the day of NTS injection until the day of mouse sacrifice.

### Statistics.

All data are expressed as mean ± SEM. For comparisons between 2 groups with normally distributed data, statistical significance was assessed using 2-tailed unpaired Student’s *t* test. For comparisons between 2 groups with non-normally distributed variables, a Wilcoxon’s test was used. Comparison among groups was performed with 1-way ANOVA, followed by the Student-Newman-Keuls test or Kruskal-Wallis test.

### Study approval.

All animal experiments were approved by the IACUC at the Durham VA Medical Center and carried out in accordance with the *Guide for the Care and Use of Laboratory Animals* (National Academies Press, 2011). The study using human kidney biopsy specimens was approved by the Institutional Review Committee of Nanjing University Medical School (Nanjing, China).

## Author contributions

JR, YX, XL, LW, SI, and SDC designed the experiments. JR, YX, XL, LW, and SI performed the experiments. JR, GH, TS, JRP, RFS, and SDC analyzed and interpreted the data and results. JR and SDC wrote the manuscript. All authors reviewed the final version of the manuscript.

## Supplementary Material

Supplemental data

## Version 1 08/09/2021

Electronic publication
