# Supplementary material for: Twist1 in podocytes ameliorates podocyte injury and proteinuria by limiting CCL2-dependent macrophage infiltration
Source: JCI Insight. 2021 Aug 9;6(15):e148109. doi: 10.1172/jci.insight.148109 (PMC8410065; doi:10.1172/jci.insight.148109)
Supplement: Supplemental data [file jciinsight-6-148109-s019.pdf]

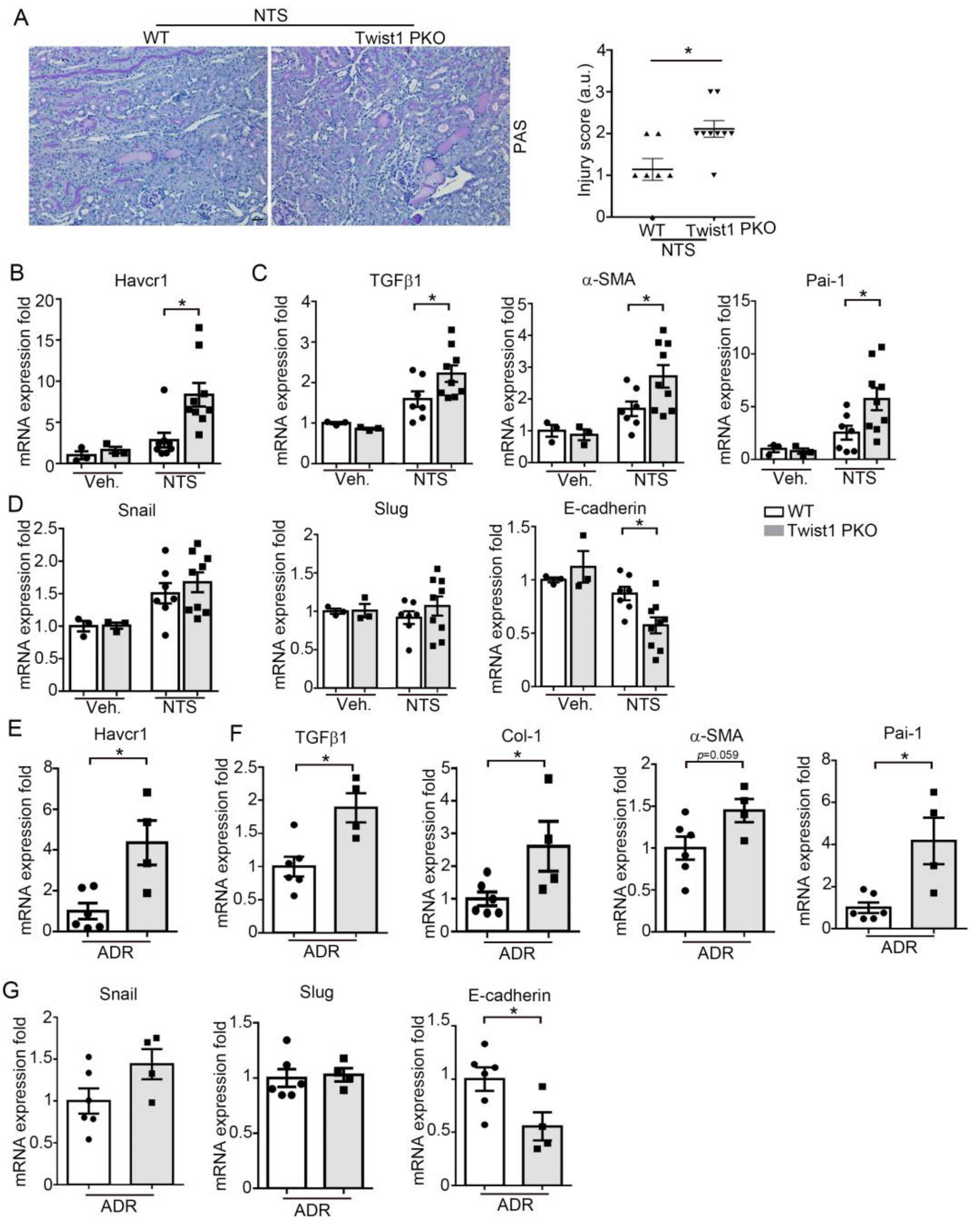

Supplementary Figure S1. Twist1 deletion in podocytes instigates renal tubular injury and expression of pro-fibrotic mediators. (A) Representative micrographs of periodic acid-Schiff (PAS) staining showing renal tubular injury and pathology scores from WT and Twist1 PKO mice (n=7-9, Kruskal-Wallis test). (B-D) Renal mRNA expression of *Havcr1* gene that encodes kidney injury molecule-1 (Kim-1) (B), mRNA for genes encoding TGFβ1, α-SMA, PAI-1 (C), Snail, Slug and E-cadherin (D) from WT and Twist1 PKO mice with NTS injury (n=3-9, Student-Newman-Keuls test). (E-G) The mRNA levels for Havcr1 = Kim-1 (E), TGFβ1, Col-1, α-SMA, PAI-1 (F), Snail, Slug and E-cadherin (G)

from WT and Twist1 PKO mice after ADR exposure (n=4-6, t test). Data represent the mean ±SEM. *$P<0.05$, Veh., vehicle; NTS, nephrotoxic serum; ADR, adriamycin, Twist1 PKO, Pod-Cre Twist1$^{fl/fl}$; WT, wild-type. Scale bar, 40μm.

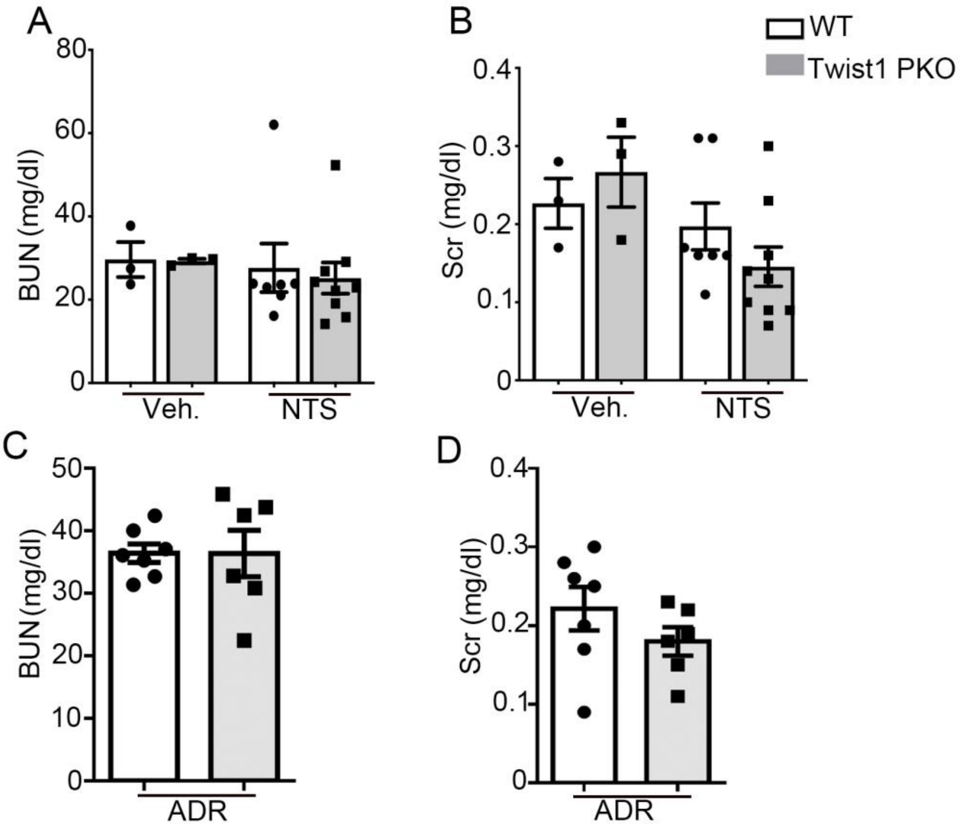

Supplementary Figure S2. Blood urea nitrogen (BUN) and serum creatinine (Scr) of WT and Twist1 PKO mice after NTS (A and B) or ADR (C and D) exposure. Data represent the mean ±SEM. Veh., vehicle; NTS, nephrotoxic serum; ADR, adriamycin, Twist1 PKO, Pod-Cre Twist1$^{fl/fl}$; WT, wild-type.

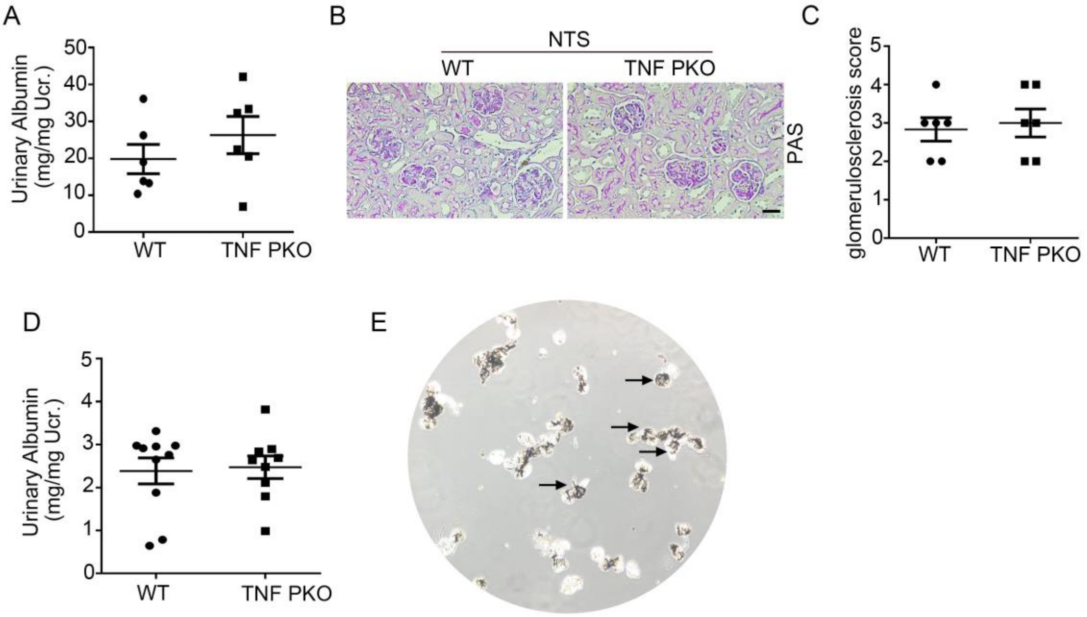

Supplementary Figure S3. TNF deletion in podocytes does not affect albuminuria and glomerular disease progression. (A) Urinary albumin concentration in NTS-exposed WT and TNF PKO mice (n=6, t test). (B) Representative images of kidney sections from WT and TNF PKO after NTS treatment. (C) Kidney glomerulosclerosis score in groups (n=6, t test). (D) Urinary albumin concentration in ADR-exposed WT and TNF PKO mice (n=9-10, t test). (E) Representative images of isolated glomeruli. Arrows indicated iron beads within glomeruli. TNF PKO, Pod-Cre; TNF$^{fl/fl}$; WT, wild type. Scale bar, 40 μm.

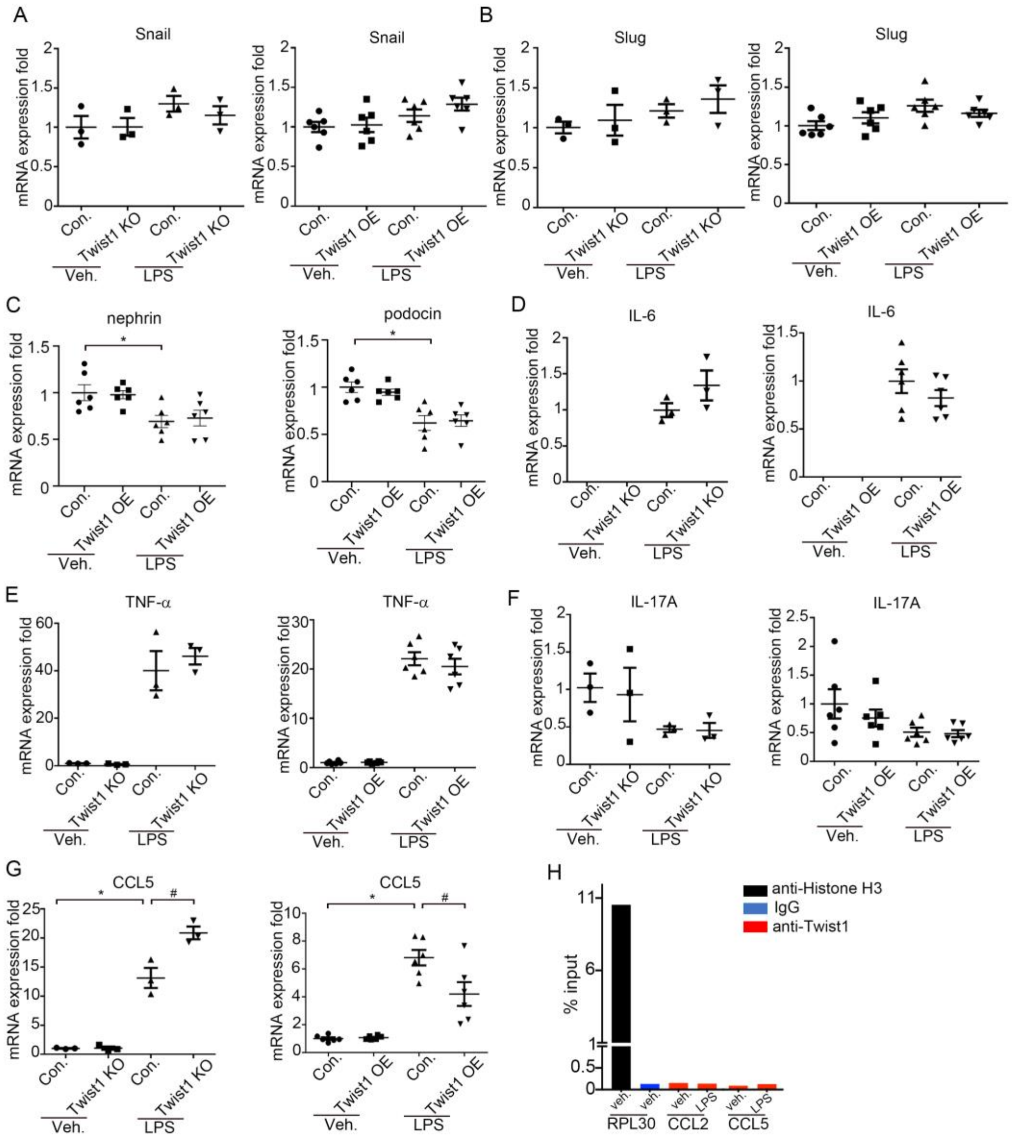

Supplementary Figure S4. CCL5 expression is regulated by Twist1 in podocytes under LPS treatment. (A-G) mRNA expression for Snail (A), Slug (B), nephrin and podocin (C), IL-6 (D), TNF-a (E), (IL-17A) (F), and CCL5 (G) in podocytes cultured in vitro as indicated under different treatments (n=3-6, Student-Newman-Keuls test). (H) qPCR analysis of ChIP performed against Histone H3 (positive control, black), IgG (negative control, blue) and Twist1 (red). Percentage input of the target regions in the promoters of CCL2, CCL5, and the housekeeping gene RPL30. Data represent the mean ±SEM. **P*<0.05, #*P*<0.05. con., control plasmid; Twist1 KO, Twist1 knock out; Twist1 OE, Twist1-overexpressing plasmid; veh., vehicle; LPS, lipopolysaccharide.

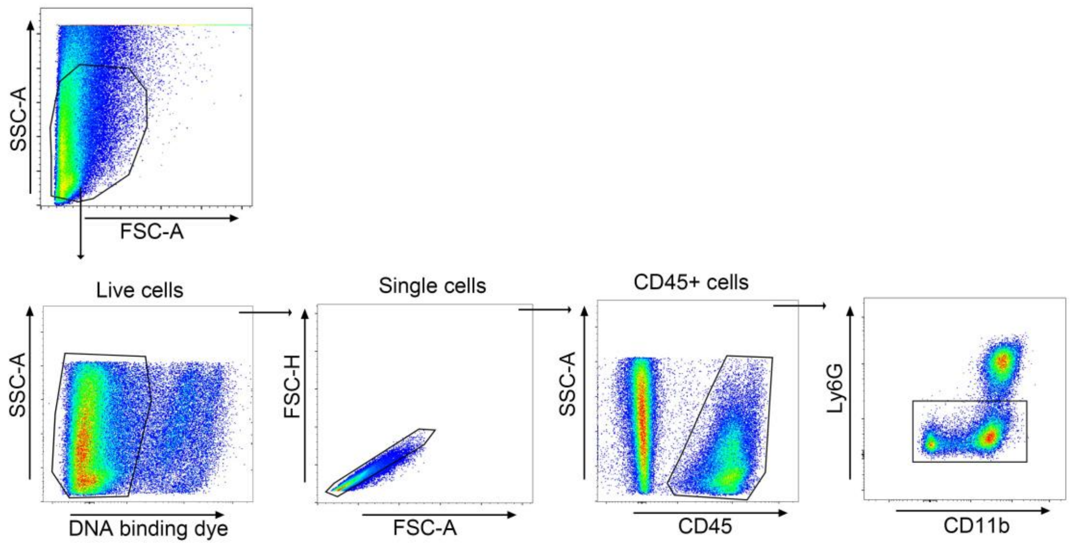

Supplementary Figure S5 The gating strategy for excluding cell debris and analyzing viable myeloid cells in NTS-exposed kidneys. FSC, forward scatter; SSC, side scatter.

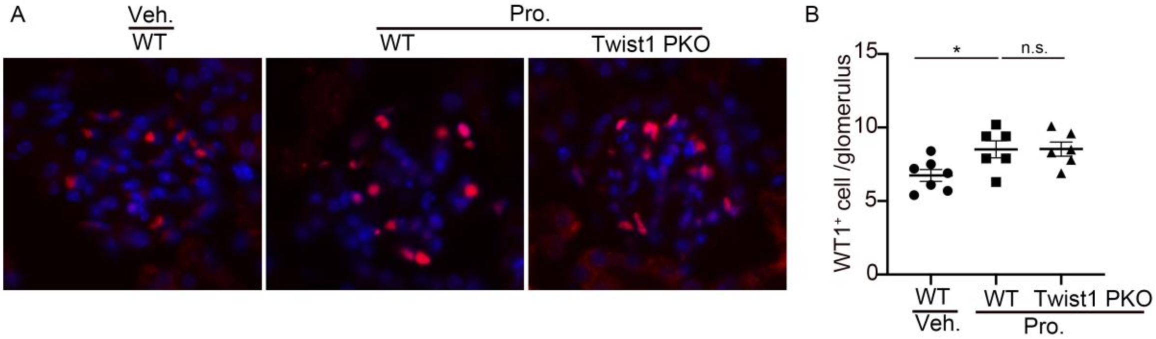

Supplementary Figure S6

(A-B) CCL2 inhibition restored and equalized WT1-positive cell number between the WT and Twist1 PKO cohorts after NTS injury (n=6, Student-Newman-Keuls test). Data represent the mean ±SEM. *P<0.05, n.s., not significant. Pro. propagermanium; veh., vehicle, Twist1 PKO, Pod-Cre; Twist1$^{fl/fl}$, WT, wild-type.
